# Anatomical Study of the Palmaris Longus Muscle and Its Clinical Importance

**DOI:** 10.3390/diagnostics15030304

**Published:** 2025-01-27

**Authors:** Abdul-Malik Al Risi, Sara Al Busaidi, Hamood Al Aufi, Lubna Al Hashmi, Srinivasa Rao Sirasanagandla, Srijit Das

**Affiliations:** Department of Human & Clinical Anatomy, College of Medicine & Health Sciences, Sultan Qaboos University, Al Khoud, Muscat 123, Oman; s133222@student.squ.edu.om (A.-M.A.R.); sara.busaidi55@gmail.com (S.A.B.); s133320@student.squ.edu.om (H.A.A.); s129223@student.squ.edu.om (L.A.H.); srinivasa@squ.edu.om (S.R.S.)

**Keywords:** anatomy, grip strength, muscle, Oman, palmaris longus, variation

## Abstract

**Background:** The palmaris longus (PL) is a long, thin muscle in the forearm’s flexor compartment, known for its variations. The present study aimed to study the PL muscle in Omani medical students and its correlation with grip strength. **Methods:** A cross-sectional prospective study was conducted among 240 medical students (120 males, 120 females) at Sultan Qaboos University, comprising 480 upper limbs. Participants were between 18 and 25 years old and had no history of forearm surgery. We considered 480 upper limbs irrespective of left or right side, and our main aim was to compare the grip strength according to the presence or absence of PL. We assessed the presence of the PL muscle using Schaeffer’s Test and Pushpakumar’s test and measured the grip strength using the CAMRY digital hand dynamometer. The data were analyzed using descriptive statistics, Chi-square tests, and independent *t*-tests. **Results:** The PL was present in 92.50% of the subjects (irrespective of side), with a higher prevalence in the females. The PL was absent in 24 (10.00%) males and 12 (5.00%) females, respectively. Average grip strength was 21.4 ± 5.25 kg in females and 40.92 ± 7.79 kg in males without considering PL presence or absence. If PL was present, then the mean grip strength was 30.84 ± 11.71 kg, and if the PL was absent, then the mean grip strength was 35.05 ± 12.44 kg. However, the *p*-value did not show any significant differences (*p* = 0.057). **Conclusions**: The PL is highly prevalent, especially in females, and its absence does not significantly affect grip strength. Hence, PL may be used for successful reconstructive surgeries without affecting hand function.

## 1. Introduction

The palmaris longus (PL) is a long and thin muscle that originates from the medial epicondyle of the humerus, the deep fascia, and the intermuscular septa next to them. It traverses next to the flexor carpi radialis and turns into a tendon in the middle of the forearm [1]. It has a long tendon that traverses superficially to the flexor retinaculum and continues to the palm as a palmar aponeurosis [1]. The anterior ulnar recurrent artery gives a small branch to the muscle belly, and the median nerve innervates the PL. The PL muscle flexes the wrist joint and tightly holds the skin and fascia of the hand against shearing forces from the side [1].

The muscle may not be present on one or both sides. Published research reports reveal that the PL muscle can be double, triple, reversed, or present as a digastric muscle with different points of insertion [2,3]. While its action in carpal flexion is restricted, the PL is best suited for use in plastic and reconstructive surgeries because its removal does not result in any functional loss in the forearm and hand, and it is approved to be used in pulley reconstruction, ocular defects, and ligament reconstruction [3,4,5,6,7]. The tendon of the PL has been used in various reconstructive surgeries.

There are several methods of physical examination, such as Schaeffer’s test, Pushpakumar’s test, Thomson’s test, and Mishra’s test. These simple, non-invasive techniques have been widely applied in various research studies to evaluate population-level differences and explore the clinical implications of the PL and its absence [8,9,10,11,12]. The absence of the PL may vary across different regions and ethnic groups and no particular reasons are given for such. A meta-analysis reveals a worldwide absence rate of 20.25%, with greater absence rates in the Arab Middle Eastern population (41.7%), Europe (24.7%), and North America (15.5%) compared to Asia (6.8%). When researchers used the Schaeffer test, the absence rate in the Arab Gulf region was 12.7%, whereas the ultrasonic approach indicated 5.7% [13,14,15]. While two studies from the Arab Gulf region were meta-analyses [13,14], only one original research study, with a sample size of 158, looked into the prevalence in the age group of 19–24 years [15]. Hence, we aimed at a larger sample size of 480 upper limbs, with the age of subjects between 18–25 years. Although past studies have been performed at other places, to the best of our knowledge, no study from the Arab Gulf region correlated the PL with grip strength with the described method.

The main aim of the present study was (i) to examine the association between the presence of the PL and gender and (ii) to observe its effect on grip strength in the adult population of Oman, as there is a paucity of published literature in the Sultanate of Oman regarding the PL and its variations.

## 2. Materials and Methods

This cross-sectional study was prospectively conducted at the College of Medicine and Health Sciences at Sultan Qaboos University (SQU), focusing on the examination of the presence of the palmaris longus (PL) muscle and grip strength. All subjects were Omani medical students. Undergraduate medical students from SQU aged between 18–25 years were taken for the study. The undergraduate medical students were taken as they were easy to recruit. Only those subjects with no prior forearm surgery and those who provided informed consent were recruited for the study. Participants who were not medical students at SQU, those who were under 18 or over 25 years of age, had a prior history of forearm surgery, or who did not provide informed consent, were excluded from the study.

The sample size was determined using nMaster software version 2.2, based on a single proportion of 0.88 observed in an earlier study [2]. With an absolute precision of 3% and a 95% confidence level, the necessary sample size was calculated to be 451 wrists, which was rounded to approximately 230 adults to ensure robust statistical validity. Consequently, 240 medical students from the College of Medicine and Health Sciences, equally divided by gender (120 males and 120 females), were enrolled, resulting in the examination of 480 upper limbs.

Two specific tests were employed to determine the presence of the PL: Schaeffer’s test and Pushpakumar’s test. Before the start of the procedures, standardization and strategies to minimize bias were kept in mind and the investigators were trained accordingly.

**Figure 1:**
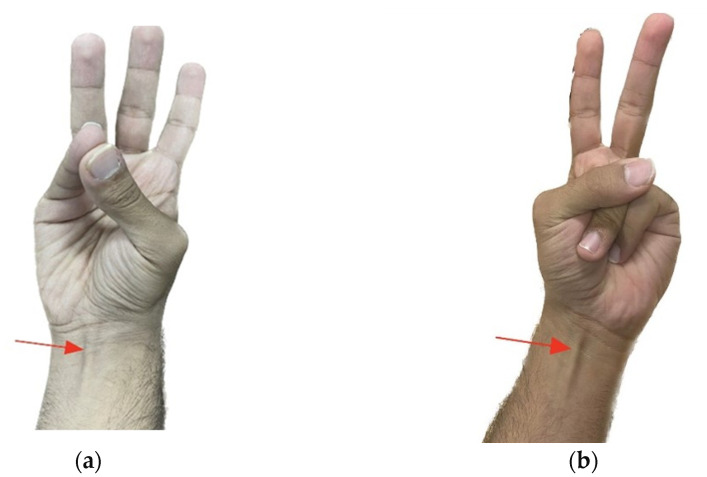
Tests employed for palmaris longus: (**a**) Schaeffer’s test; (**b**) Pushpakumar’s test. Red arrow shows the PL tendon.

In Schaeffer’s test, participants flexed their wrists and formed fists to palpate the tendon in the wrist area (Figure 1).

If the tendon was not visible, Pushpakumar’s test was conducted, during which the participants extended their index and middle fingers while flexing the wrist and other fingers, with the thumb fully opposed and flexed (Figure 1). This approach ensured a thorough assessment of the PL muscle’s presence.

**Figure 2:**
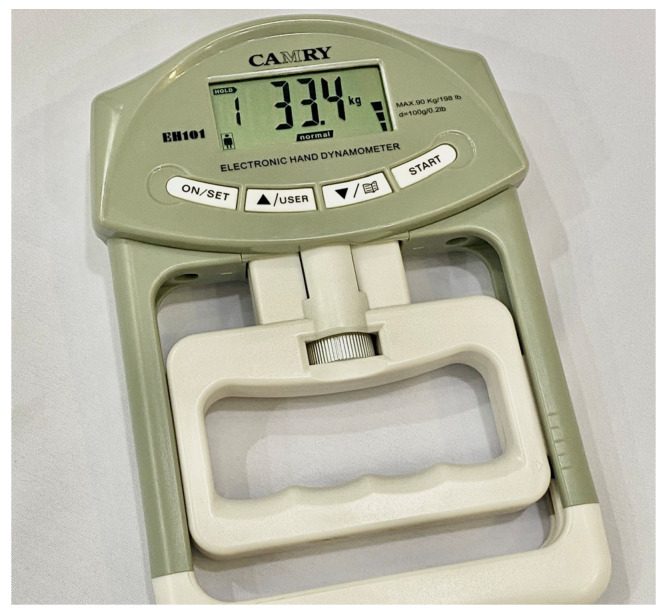
CAMRY digital hand dynamometer grip strength measurement meter auto-capturing electronic hand grip power.

The grip strength was measured using the CAMRY digital hand dynamometer grip strength meter (Figure 2), which was calibrated before each session. Participants sat with their feet flat on the ground, their backs held straight and their elbows flexed at 90° and close to the body, with wrists in the neutral position. The participants squeezed the dynamometer with their hands as hard as possible, and the device recorded the peak grip strength.

The data collection was carried out with participants providing informed consent after being briefed on the study’s objectives. Data analysis was performed using SPSS software, version 25, employing descriptive statistics and the Chi-square test to examine the association between the presence of the PL and gender. An independent *t*-test compared grip strength across genders and between individuals with and without the PL. Statistical significance was set at a *p*-value of ≤0.05.

The findings of the study are presented in graphs and tables to show the prevalence of the PL in the Omani population.

Ethical approval for this study was obtained from the Medical Research and Ethics Committee (MREC) of the College of Medicine and Health Sciences (COMHS), with ethical number MREC#3105. Participants’ data was coded and secured with encryption and password protection to ensure confidentiality and safety.

## 3. Results

### 3.1. Demographic Characteristics

The study included an equal number of male and female participants; each group comprised 120 individuals, which represented 50% of the total study population (*N* = 240). The mean age of the male participants was 21.16 years (SD ± 1.74), slightly higher than that of the female participants, who had a mean age of 20.67 years (SD ± 1.06). Overall, the combined mean age of the study cohort was 20.91 years (SD ± 1.46) (Table 1).

### 3.2. Prevalence of PL

In the Omani population studied, the overall prevalence of the PL was 92.50%, with the muscle present in 444 out of 480 total wrist assessments and its absence observed in 36 wrists, accounting for 7.50% of the assessments. Gender-specific statistics revealed the following subtle differences: among males, the PL was present in 216 out of 240 wrists, corresponding to a prevalence rate of 90.0%, with its absence noted in 24 wrists (10.00%). Conversely, the prevalence was slightly higher among females, with the PL present in 228 out of 240 wrists, which translated to a 95.0% prevalence rate. Only 12 wrists (5.00%) in the female cohort were without the PL. Despite these differences, the statistical analysis showed a *p*-value of 0.386 when applying the Chi-square test, indicating that the difference in PL prevalence between genders was not statistically significant (Table 2).

Table 3 illustrates the variations in the prevalence of the PL between the right and left hands of all the participants. For the right hand, out of 240 assessments, the PL was present in 219 cases, translating to a prevalence rate of 91.25%. Conversely, the absence of PL in the right hand was noted in 21 cases, or 8.75%. In comparison, the left hand exhibited a slightly higher prevalence, with PL present in 225 of the 240 assessments, which resulted in a prevalence rate of 93.75%. Only 15 cases in the left hand showed an absence of PL, corresponding to a 6.25% absence rate. These findings suggest a marginally higher prevalence of PL in the left hand compared to the right hand. However, with a *p*-value of 0.057 when applying the Chi-square test, the statistical analysis suggests that the difference in PL prevalence between the right and left hands did not show any statistical significance.

**Figure 3:**
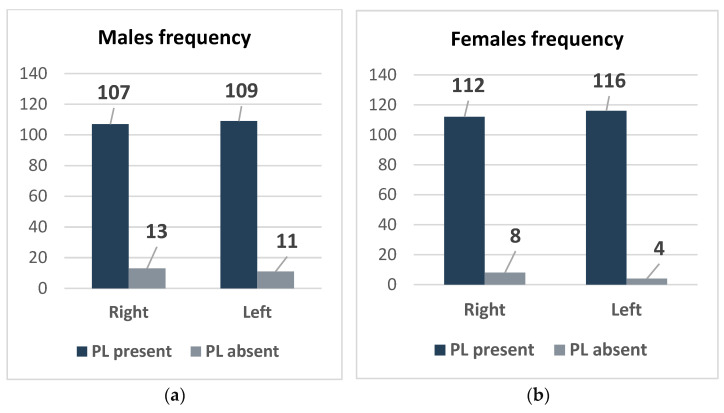
Frequency of PL association with right and left hands in males and females. (**a**) Frequency of PL associated with right and left hands in males; (**b**) frequency of PL associated with right and left hands in females.

Figure 3a,b depict the prevalence of the PL across different hands and genders. Regarding the females, presence of the PL was noted in 112 right hands and 116 left hands, with a slightly higher prevalence in the left hand, whereas it was absent in 8 right hands and only 4 left hands, indicating a strong presence with minimal absence. In contrast, males displayed a PL in 107 right hands and 109 left hands, with a slightly increased frequency in the left hand. However, the absence was more pronounced among males, with 13 right hands and 11 left hands lacking a PL. Overall, the data indicate that both males and females had a higher PL prevalence in the left hand, with females showing a greater overall presence and fewer absences of a PL than males.

### 3.3. Grip Strength

Table 4 details the grip strength differences across genders. The overall mean grip strength for all participants was 31.16 kg, with a standard deviation of 11.81 kg, ranging from a minimum of 8.3 kg to a maximum of 64.5 kg. When disaggregated by gender, females had a mean grip strength of 21.4 kg, with a standard deviation of 5.25 kg, with their grip strength ranging from 8.3 to 38.6 kg. Males demonstrated a notably higher mean grip strength of 40.92 kg, with a standard deviation of 7.79 kg, ranging from 12.0 kg to the maximum observed of 64.5 kg. The significant relationship in grip strength between the genders was statistically confirmed with a *p*-value of less than 0.001 when applying *t*-tests; to be exact, 2.42 × 10^−115^, which indicates a highly significant difference and reflects distinct gender-based physiological characteristics in muscle strength.

While examining grip strength with respect to the presence or absence of the PL (Table 5), it was observed that the participants without a PL, numbering 36, had a mean grip strength of 35.05 kg, which was higher than that of participants with a PL. The standard deviation for the PL absent group was 12.44 kg, and their grip strength varied between 12.6 kg and 58.0 kg. On the other hand, a significantly larger sample of 444 participants with a PL showed a mean grip strength of 30.84 kg, with a standard deviation of 11.71 kg and a range from 8.3 kg to 64.5 kg. Although individuals without a PL appeared to have greater mean grip strength, the statistical analysis with *t*-tests showed a *p*-value of 0.057, which indicates that the difference in grip strength based on the presence of PL was not statistically significant, as it marginally exceeded the conventional cutoff of 0.05.

Regarding females, the analysis revealed no significant difference in grip strength between individuals with and without a PL. The mean grip strength for females with a PL was 21.46 ± 5.14 kg, while for those without a PL, it was 21.04 ± 7.41 kg. The range of grip strength was 8.3–38.6 kg for females with a PL and 12.6–32.2 kg for those without. Statistical analysis with a *t*-statistic of 0.19 and a *p*-value of 0.85 indicates no significant association between the presence of a PL and grip strength in females (Table 6).

Regarding males, the analysis revealed no significant difference in grip strength between individuals with and without a PL. The mean grip strength for males with a PL was 40.75 ± 7.71 kg, while for those without a PL, it was 42.05 ± 7.47 kg. The range of grip strength was 12.0–64.5 kg for males with a PL and 29.8–58.0 kg for those without. Statistical analysis with a *p*-value of 0.43 indicates no significant association between the presence of a PL and grip strength in males (Table 7).

## 4. Discussion

Compared to an earlier study in the Arab Gulf region, where no statistical basis was stated for the calculation of the necessary sample size using absolute precision and confidence level, we used the nMaster software version 2.2 to calculate the sample size. It is pertinent to mention that our sample size was higher than that of an earlier study conducted in the Arab Gulf region [15].

The overall high prevalence of PL present in our study cohort (92.50%) was higher than the worldwide prevalence according to a meta-analysis that reported 79.75% presence [13]. Our study found a higher prevalence of PL being present compared to another study conducted in Saudi Arabia (84.90%) [16], but not as high as reported in other parts of the world such as Malaysia (93.60%) [17]. The study in Saudi Arabia had a lower sample size (*N* = 331) [16] compared to ours (*N* = 480). Another Indian study observed the PL to be absent unilaterally in 19.48% of males and 14.2% of females, with the unilateral absence of a PL tendon being 16.9% and the bilateral absence being 3.3% [12]. Research reports have shown weak flexor digitorum superficialis with absent PL tendon to be insignificant in the female population [12]. Another study in Nigeria found that unilateral absence was higher in females (15.1%) compared to males (11.2%) [18]. The higher prevalence of PL among Omani females compared to males, and in the left hand compared to the right, was intriguing. If we compare our results to another study in the Arab Gulf region, i.e., in Bahrain, we find that the study in Bahrain reported the absence of a PL to be more frequent in females [15], whereas, in our study, it was more frequently absent in males. Although these differences were not statistically significant (*p*-values > 0.05) in the present study, there may be underlying biological or genetic mechanisms that may have influenced PL development. With advancement in age, agenesis of the PL may be more common, but in our study, the subjects were young medical students. There is a paucity of published literature on ethnic heterogeneity.

A recent research study reported that it is the geographical origin rather than ethnicity that determines the presence of PL [19]. The same study reported that environmental factors are more important than genetic factors for the presence of a PL [19]. Environmental and genetic factors may be closely linked, as reported by Cohen et al. (2023) [19]. As the first study of its kind in Oman, we aimed to investigate the prevalence of the PL among Omanis and determine its impact on grip strength, with special attention to gender-specific variations.

The results of our study provide a detailed overview of the prevalence of PL and the variability in grip strength among Omani medical students. Our study revealed differences in the prevalence of the PL and grip strength between males and females, as well as between the right and left hands. This suggests that there might be genetic or developmental factors influencing who possesses the PL and who does not, and how strong their grip is. These results have practical implications for diagnostic and surgical practices and provide insights into grip strength that could impact job selection processes requiring strong grip capabilities. The study offers a valuable reference for future research and ensures that the outcomes are both statistically significant and practically relevant in medical and occupational settings. Our subjects were young and were not in any professional job other than studying medicine.

The results of the present study offer valuable insights for surgeons on the use of the PL, which has implications for surgical techniques, particularly in forearm reconstructive surgeries, where an understanding of anatomical variation is critical. Many surgeons feel that the PL tendon could be considered the first choice as a donor tendon because it has the required length, diameter, and availability [20]. Another advantage of using the PL tenon is that it causes no functional deformity [21]. There are reports of the muscle tendon being used in surgeries involving lip augmentation or escalation [22], ptosis correction [23], and surgical correction of facial paralysis [21]. The free flap technique has been effectively used to reconstruct the lower lip with a functioning oral sphincter [24].

The significant differences in grip strength between genders were consistent with the European literature that reported higher muscular strength in males [25]. This difference was profound and statistically significant in our study, highlighting strong gender-based physiological differences possibly rooted in muscle fiber composition [26] or hormonal differences despite a notable difference in means, suggesting that the PL is not a significant determinant of grip strength in this population, as the tests showed a non-significant *p*-value.

Hand grip is important in various sports. Sports such as tennis, badminton, and squash are considered sustained dominant-sided cylindrical grip sports, while on the other hand, rowing and canoeing are considered sustained two-handed cylindrical grip sports. In hockey and cricket, sustained grip is important for stroke play. Intermittent grip sports are seen in golf. Researchers held the view that in both elite and non-elite athletes, the performance of the PLM was found to be significantly higher in individuals who participated in sustained grip sports while being compared to intermittent grip sports. Interestingly, in elite athletes who participated in dominant-handed and two-handed cylindrical grip sports, it was observed that the presence of the PLM was greater on the dominant side compared to that of non-elite sportspersons playing the same sports [27]. The bilateral occurrence of the PLM was also higher in elite athletes [27]. Also, there are reports by other researchers that showed that the absence of PL was not associated with a decrease in grip or pinch strength [28]. The difference was not statistically significant, and no direct correlation was found between the PL muscle and performance in tennis-specific activities or between the PL muscle and overall athletic performance [29,30]. Admittedly, we did not obtain a history of any sports activities from our subjects.

The PL acts as a synergist in the movement of the thumb, and this was proven by previous research, which showed synergistic action between PL and the abductor pollicis brevis muscles of the thumb [31]. The PL can transmit force to the thenar eminence of the palm. Surgical opponensplasty has been beneficial in treating function loss in thenar paralysis [32]. Interestingly, there are reports that the PL may provide up to 15 cm for grafts and hence, may be chosen easily [33]. The muscle has been used effectively for pulley reconstructions. Detailed morphological studies on muscle cross-sectional area may reveal the maximal force production capacity. Future studies can be performed on a larger sample size. Recent reports showed successful arthroscopic transosseous repairs of the triangular fibrocartilage complex when the PL tendon was used [34].

Interestingly, surgeons treating carpal tunnel syndrome should be aware of uncommon etiologies. An interesting case of a 40-year-old woman diagnosed with a rupture of the distal PL resulting in acute carpal tunnel syndrome has been reported [35]. One of the reasons attributed to this condition was subclinical tendinitis of the palmaris longus tendon due to repetitive clapping.

### 4.1. Limitations

The study may have several limitations that warrant consideration when interpreting its findings. Firstly, the sample was composed of medical students from SQU, limiting the diversity of the sample and potentially affecting the generalizability of the results to the entire Omani population. This selection bias might overlook the variations in anatomical and physiological characteristics present across different demographics within the nation. Secondly, the methodology for measuring grip strength may also introduce some inconsistencies. The grip strength readings could vary depending on the time of day the measurements were taken and the participant’s level of physical activity before the assessment. Such variations could lead to fluctuations in the results, potentially obscuring true associations between the presence of the PL and grip strength. These factors should be carefully considered when applying the study’s findings in clinical or academic settings. We did not consider the dominant side of each subject. We admit that it could affect the grip strength.

### 4.2. Recommendation

For future research, it would be beneficial to study a more diverse group of people to see if these results hold across different populations. We believe that adding ultrasonography to the research methods could help with knowing the exact measurements of the PL, which are needed for various transplant surgeries. The anatomical relationship of the median nerve traversing deep to the PL and flexor retinaculum at the wrist can be well interpreted in ultrasonographic findings and may be beneficial in treating any nerve compression. Genetic study of the muscle variants could be planned in the future. Prior anatomical knowledge regarding the presence of the PL may have a better outcome.

## 5. Conclusions

The majority of the studies on PL have been performed on cadavers. Many individuals are not aware if they possess a PL or not. Unless daily activity is hampered, an individual may not face any difficulty in performing any particular task. The present study showed that the PL was present in 92.50% of the participants, with a higher occurrence in females and more frequently on the left upper limb. Interestingly, there is no significant impact of the absence of the PL on grip strength. The absence of the PL not affecting grip strength implies that there are several other factors influencing grip strength. Hence, using the PL for reconstructive surgeries may not cause any functional disorder or hamper daily activity in any profession. It will be interesting to note any changes due to the absence of the PL in individuals in different professions.

## Figures and Tables

**Table 1:** Demographic characteristics of participants.

	Number of Participants	Mean Age (Years)
Male	120 (50%)	21.16 (SD ^1^ ± 1.74)
Female	120 (50%)	20.67 (SD ± 1.06)
overall	240	20.91 (SD ± 1.46)

^1^ SD: standard deviation.

**Table 2:** Variation of PL in males and females.

Gender	PL Present	PL Absent	Total
Male	216 (90.00%)	24 (10.00%)	240 (100.00%)
Female	228 (95.00%)	12 (5.00%)	240 (100.00%)
Total	444 (92.50%)	36 (7.50%)	480 (100.00%)

*p*-value: 0.386.

**Table 3:** Right and left hand variations of PL.

Hand	PL Present	PL Absent	Total
Right Hand	219 (91.25%)	21 (8.75%)	240 (100.00%)
Left Hand	225 (93.75%)	15 (6.25%)	240 (100.00%)
Total	444 (92.50%)	36 (7.50%)	480 (100.00%)

*p*-value: 0.057.

**Table 4:** Grip strength comparison according to gender.

Variable	Females	Males	All Participants
Number	240	240	480
Mean ± SD ^1^	21.4 ± 5.25	40.92 ± 7.79	31.16 ± 11.81
Min–Max	8.3–38.6	12.0–64.5	8.3–64.5

^1^ SD: standard deviation. *p*-value: < 0.001 (specifically, 2.42 × 10^−115^).

**Table 5:** Grip strength comparison according to PL presence or absence.

Variable	PL Absent	PL Present	Total
Number	36	444	480
Mean ± SD ^1^	35.05 ± 12.44	30.84 ± 11.71	31.16 ± 11.81
Min–Max	12.6–58.0	8.3–64.5	8.3–64.5

^1^ SD: standard deviation; *p*-value: 0.057.

**Table 6:** Grip strength comparison in females according to PL presence or absence.

Variable	PL Absent	PL Present	Total
Number	12	228	480
Mean ± SD ^1^	21.04 ± 7.41	21.46 ± 5.14	21.40 ± 5.25
Min–Max	12.6–32.2	8.3–38.6	8.3–38.6

^1^ SD: standard deviation; *p*-value: 0.85.

**Table 7:** Grip strength comparison in males according to PL presence or absence.

Variable	PL Absent	PL Present	Total
Number	24	216	480
Mean ± SD ^1^	42.05 ± 7.47	40.75 ± 7.71	40.92 ± 7.79
Min–Max	29.8–58.0	12.0–64.5	12.0–64.5

^1^ SD: standard deviation; *p*-value: 0.43.

## Data Availability

The data presented in this study are available on request from the corresponding author. The findings relate to the subjects whose privacy may be needed. Ethical approval allowed us to perform the study on the basis of confidentiality and privacy.
